# *Akkermansia muciniphila* and herbal medicine in immune-related diseases: current evidence and future perspectives

**DOI:** 10.3389/frmbi.2024.1276015

**Published:** 2024-02-05

**Authors:** Xue Ding, Peng-Fei Meng, Xiu-Xia Ma, Jing-Yu Yue, Liang-Ping Li, Li-Ran Xu

**Affiliations:** ^1^ Department of Medical, The First Affiliated Hospital of Henan University of Chinese Medicine, Zhengzhou, China; ^2^ Department of the First Clinical Medical College, Henan University of Chinese Medicine, Zhengzhou, China; ^3^ Department of AIDS Clinical Research Center, Henan University of Chinese Medicine, Zhengzhou, China; ^4^ Department of Graduate, Henan University of Chinese Medicine, Zhengzhou, China; ^5^ Department of Zhongjing Academy of Traditional Chinese Medicine, Henan University of Chinese Medicine, Zhengzhou, China

**Keywords:** *Akkermansia muciniphila*, immune-related disease, inflammation, herbal medicine, immune system

## Abstract

*Akkermansia muciniphila* is considered the “paradigm for next-generation beneficial microorganisms” and has been reported to help alleviat immune-related diseases. Evidence shows that herbal medicine can treat disease by regulating the abundance of *A. muciniphila*. Recent studies have revealed a link between *A. muciniphila* and immune-related diseases. Here, we systematically reviewed the association between *A. muciniphila*, herbal medicine, and immune-related diseases (including inflammatory bowel disease, human immunodeficiency virus, cancer immunotherapy, and immune-related liver injury). We also summarize the potential mechanisms of action of *A. muciniphila* and offer perspectives for future studies.

## 1 Introduction

Immune-related diseases (IRDs) prevalence widely around the world, with an increasing incidence of autoimmune disease in recent years (Brodin, 2022). The mechanisms of some IRDs might be related to alterations in immune–microbe interactions and impaired immune function, leading to recurrent infections, chronic inflammation, and nutritional deficiency. The gut microbiota affects host health, and changes in the abundance of organisms within the gut microbiota have been linked with cancer, type 2 diabetes, obesity, intestinal bowel disease, and neurodegenerative diseases (Derosa et al., 2022; Bi et al., 2023; Ghotaslou et al., 2023).

The intestine is predominantly colonized by four bacterial phyla: Firmicutes, Bacteroidetes, Actinobacteria, and Verrucomicrobia (Bibbò et al., 2017). *Akkermansia muciniphila* is the only representative of the Verrucomicrobiota phylum and is highly effective at mucin degradation and considered the “paradigm for next-generation beneficial microorganisms”. Since *A. muciniphila* was discovered and characterized two decades ago, changes in this commensal bacterium have led to it being linked to multiple diseases in humans, such as IRDs and metabolic disorders (Cani et al., 2022). *A. muciniphila* shows alterations in abundance (in some cases a significant reduction) in patients with IRDs (Scher et al., 2015; Tan et al., 2018; Stoll et al., 2023; Zhao et al., 2023). These findings suggest that *A. muciniphila* may confer a clinical benefit in patients with IRDs.

Herbal medicine is gaining increasing attention worldwide because of its powerful therapeutic effects and minimal adverse effects. Herbal medicine can regulate the composition and metabolism of intestinal flora and inhibits disease by regulating the abundance of *A. muciniphila* (Feng et al., 2019; Su et al., 2020; Zhang et al., 2022b). Herbal medicine might have beneficial biogenic effects on *A. muciniphila*, and it plays an important therapeutic role by regulating the population, distribution, and metabolites of intestinal microbiota to promote its probiotic function. Herbal medicine also affects systemic immunity by increasing the abundance of *A. muciniphila* in the intestine (Barratt et al., 2022), suggesting that upregulation of *A. muciniphila* abundance may be strongly associated with IRDs, making the impact of herbal medicine on *A. muciniphila* and IRDs a promising area with potentially far-reaching implications. In this review, we will discuss the close relationship between herbal medicine, *A. muciniphila* and IRDs by examining the biological characteristics of *A. muciniphila*, the effects of herbal medicine in regulating the abundance of *A. muciniphila*, and the mechanisms underlying the role of *A. muciniphila* in IRDs.

## 2 Characteristics of *A. muciniphila*

*A. muciniphila* is an oval-shaped Gram-negative bacterium that is strictly anaerobic, non-motile, and non-endospore-forming. *A. muciniphila* can produce acetate, butyrate, and propionate in the gut as short-chain fatty acids via the process of mucin fermentation. Genome analysis of *Akkermansia* revealed four phylogroups (AmI, AmII, AmIII, and AmIV) (Kirmiz et al., 2020). Phylogroups AmIV and AmII outcompete AmI strains in antibiotic-treated mice, AmIII is predominant in the Chinese population, AmIV is predominant in Western populations, while AmI strains are most prominent in infants, children, and adolescents (Becken et al., 2021; Luna et al., 2022). *A. muciniphila* was initially considered a strictly anaerobic bacterium, but one study found that it could survive under microaerophilic conditions to produce additional energy relative to its oxygen sensitivity (Ghaffari et al., 2022). Phenotypes of *A. muciniphila* are resistant to gentamicin, penicillin G, vancomycin, kanamycin, streptomycin, tetracycline, and ciprofloxacin (Filardi et al., 2022; MaChado et al., 2022). One study found that in healthy middle-aged and older adults in southwest China, the abundance of *Akkermansia* positively correlated with IgA levels and the percentage of CD8+ T cells, and negatively correlated with the percentage of CD4+ T cells and the CD4+/CD8+ ratio, indicating that the intestinal flora correlates to some extent with immunity (Shen et al., 2018).

*A. muciniphila* affects the composition of immune cells and enhances immune regulation by regulating pleiotropic cytokines, including interferon (IFN)-γ, tumor necrosis factor (TNF)-α, Th17, interleukin (IL)10, IL33, and IL4, with multiple immunomodulatory effects (Derrien et al., 2010; Li et al., 2019; Chen et al., 2020; Katiraei et al., 2020). Preliminary evidence from preclinical studies has revealed the immune-regulatory potential of *A. muciniphila*. Supplementation with *A. muciniphila* increases the thickness of the intestinal mucus layer and improves the systemic immune status of mice (van der Lugt et al., 2019). *A. muciniphila* supplementation also improves immune cell chemotaxis, phagocytosis, natural killer cell activity, proliferative capacity, and reduces oxidative stress parameters and pro-inflammatory cytokines in aged mice (Cerro et al., 2022). These studies collectively support the immune-regulation potential of *A. muciniphila* and offer new directions for immune-related research.

## 3 *A. muciniphila*, herbal medicine, and immunomodulation

Probiotics are live organisms that confer a health benefit to the host when administered in adequate amounts (Cani, 2018). Probiotics play an important role in immune system modulation and the anti-inflammatory response (Plaza-Díaz et al., 2017). *A. muciniphila* alleviates persistent inflammation, mediates immunosuppression, and protects against catabolism syndrome by reshaping the microbial community. *A. muciniphila* is one of the most abundant microorganisms in humans with an immunoregulatory function (Sanders et al., 2019; Terrisse et al., 2022). Recent studies have demonstrated that a decreased abundance or lack of *A. muciniphila* is closely linked with increased inflammation in the context of multiple diseases, such as Crohn’s disease, ulcerative colitis, human immunodeficiency virus (HIV), diabetes, and obesity (Plovier et al., 2017; Depommier et al., 2019) Recently, herbal medicine has been shown to have immunomodulatory effects by regulating the abundance of *A. muciniphila* (Feng et al., 2019; Alharris et al., 2022).

*A. muciniphila* plays a key role in maintaining intestinal health by inducing intestinal adaptive immune responses and immune responses (Ansaldo et al., 2019; Wang et al., 2020). *A. muciniphila* can be used as a treatment strategy for IRDs by regulating the immune system and restoring balance to the intestinal flora (**Table 1**). Herbal medicine can be used as an immunomodulator in IRDs such as inflammatory bowel disease (IBD), rheumatoid arthritis, and hypersensitivity reactions (Teng et al., 2023). Furthermore, there is a correlation between the regulation of intestinal flora by herbal medicine and the prevention and control of IRDs (**Table 2**). The link between *A. muciniphila* and herbal medicine in IRDs has long been a topic of great interest. These studies support the interaction of *A. muciniphila* with herbal medicine in the treatment of IRDs and introduce new perspectives for future IRD research.

**Table 1:** Links between Akkermansia muciniphila and immune-related diseases.

Immune-related diseases	Model	Change in *Akkermansia* *muciniphila* abundance compared to healthy controls	Efficacy after intervention with *Akkermansia* *muciniphila*	Microbiota analysis approach	Potential mechanisms	Ref.
Systemic lupus erythematosus	Mice model	NR	Beneficial for disease	16SrRNA sequencing	Regulate cytokine levels in the circulation, restore the intestinal barrier integrity, and remodel the gut microbiome	(Guo et al., 2023)
Psoriasis	Mice model	Reduced	Beneficial for disease	16SrRNA sequencing, qPCR	Improve intestinal microenvironment and regulates the intestinal homeostasis	(Tan et al., 2018; Sonomoto et al., 2023)
Atopic dermatitis	Mice model	Increased	Beneficial for disease	Metagenomic shotgun sequencing, qPCR	Improve immune responses and the production of filaggrin in the skin and ZO-1 in the intestinal barrier.	(Hong et al., 2022; Lee et al., 2022)
Asthma	Human, Mice	Reduced	Beneficial for disease	16SrRNA sequencing, qPCR	Induce the secretion of anti-inflammatory cytokine IL-10 and prevented the secretion of pro-inflammatory cytokines like IL-12, change the microbiota composition	(Demirci et al., 2019; Michalovich et al., 2019)
Inflammatory bowel disease	Mice model	Reduced	Beneficial for disease	16SrRNA sequencing, qPCR	Protect the gut barrier function and reduce the levels of inflammatory cytokines, improve the microbial community.	(Bian et al., 2019)
Human immunodeficiency virus	Human	Reduced	NR	16S rRNA gene and whole genome shotgun metagenomic sequence	Changed the gut microbiota composition and increase the abundance of favorable anti-inflammatory bacteria	(Isnard et al., 2020; Yanavich et al., 2022)
Immune-related liver injury	Mice model	Reduced	Beneficial for disease	16SrRNA sequencing, qPCR	Enhance expression of Occludin and Tjp-1 and inhibited CB1 receptor, strengthen intestinal barriers and reduce systemic LPS level, increase microbial richness and diversity	(Wu et al., 2017)

NR, not reported; qPCR, quantitative polymerase chain reaction.

**Table 2:** Regulation of the intestinal flora by the active ingredients in herbal medicine for the treatment of immune-related diseases.

Immune-related diseases	Chinese herb	Model	Change in *Akkermansia muciniphila* abundance	Potential mechanisms	Change of indicators	Ref.
Rheumatoid arthritis	Atractylodes koreana	Mice model	Increased	Inflammatory factors ↓, imbalance of intestinal flora and SCFAs↑	synovial infiltration and vascular proliferation↓, TNF - α, IL-1, IL-1 β, IL-2, IL-6, hs-CRP ↓	(Pang et al., 2021)
Ulcerative colitis	Icariin, Coptis chinensis Franch.	Mice model	Increased	Tissue damage and inflammatory response ↓	Modulating the p-p65/p65 expression., IL-6, TNF-α, NF-κB↓	(Yang et al., 2021; Zhang et al., 2021)
Non-alcoholic fatty liver disease	Si Miao Formula, MDG, an Ophiopogon japonicus polysaccharide	Mice model	Increased	Modulate lipid-related pathways	Acly, Fas, Acc, Scd-1 ↓, pro-inflammatory cytokines (Il-1β, Nlrp-3) ↓	(Zhang et al., 2022b; Zeng et al., 2023)
Cancer Immunotherapy	Huoxue Yiqi Recipe-2	Mice model	Increased	Modulate the composition of intestinal flora	NR	(Teng et al., 2020)
Irritable bowel syndrome	Wuji Wan	Male wistar rats	Increased	Goblet cell proliferation↑, restored the mucus barrier, tight junctions↓	mucin↑, tight junction proteins Occludin, ZO-1 ↑, MLCK↓	(Chen et al., 2017)

TNF, tumor necrosis factor; IL, Interleukin; CRP, C-reaction protein; NF-κB, nuclear factor kappa-B; Nlrp-3, nucleotide binding oligomerization domain-like receptor protein 3; MLCK, myosin light-chain kinase; ZO1, tight junction protein 1; NA, not reported.

## 4 *A. muciniphila*, herbal medicine, and immune-related diseases

### 4.1 *A. muciniphila*, herbal medicine, and inflammatory bowel disease

IBD is a chronic, relapsing gastrointestinal disease that develops an inappropriate immune response to environmental factors in genetically-susceptible individuals (Wright et al., 2018). The onset of IBD is caused by combining the effects of barrier functions, intestinal microecology, and mucosal immunity (Vindigni et al., 2016). Reduced diversity of the intestinal flora leads to microbial dysbiosis, resulting in the occurrence of IBD (Marchesi et al., 2016; Thaiss et al., 2016). The abundance of *A. muciniphila* a is considered crucial in the occurrence and development of IBD. *A. muciniphil*, a promising probiotic, could protect against colitis via the regulation of the immune response. Integrative analysis of fecal metagenomes and serum metabolomes revealed that *A. muciniphila* significantly offsets the reduction in indoleacetic acid concentrations, increases the serum concentrations of indole acrylic acid, and upregulates aryl hydrocarbon receptor target genes, including CYP1A1, IL-10 and IL-22, thereby attenuating colonic inflammation (Gu et al., 2021). Additionally, the administration of *A. muciniphila* reduces the number of infiltrating macrophages and CD8+ cytotoxic T lymphocytes in the colon, which may improve colitis (Wang et al., 2020). A study of an acute colitis mice model found that gavage feeding of *A. muciniphila* decreased intestinal permeability and the level of inflammatory cytokines in serum and tissue. Analysis of 16S rDNA sequences showed that A. muciniphila induced significant gut microbiota alterations (Bian et al., 2019). Amucc_2109, an enzyme secreted by *A. muciniphila*, attenuates dextran sulfate sodium-induced colitis in mice, possibly in association with inhibition of the overexpression of inflammatory cytokines (Qian et al., 2022). In clinical practice, fecal microbiota transplantation has been used to treat IBD and has shown certain effects. Metagenomic sequencing indicated higher species diversity and higher abundance of anti-inflammatory bacteria in the fecal microbiota transplantation intervention group, including *Alistipes putredinis*, *A. muciniphila, Bifidobacterium adolescentis*, SCFAs-producing bacterium *Christensenella minuta*, and secondary bile acids-producing bacterium *Clostridium leptum*. Metabolomics analysis showed that indoleacetic acid and unsaturated fatty acids (DHA, DPA, and EPA) with anti-inflammatory effects were significantly enriched (Yang et al., 2022). These studies suggest that *A. muciniphila* could ameliorate mucosal inflammation either via microbe-host interactions, which protect the gut barrier function and reduce the levels of inflammatory cytokines, or by improving the microbial community.

Si-Ni-San (SNS) is a herbal medicine that modulates the gut microbial community and markedly inhibits inflammatory responses by improving intestinal flora dysbiosis, reducing the abundance of pro-inflammatory flora, and upregulating the abundance of anti-inflammatory species (Wang et al., 2021; Cai et al., 2023). A study exploring the potential modulatory effects of GeGen QinLian decoction on intestinal flora found that the NOD/RIP2/NF-κB signaling pathway is inhibited in the mesenteric lymph nodes and serum of mice that received fecal microbiota transplantation from mice fed GeGen QinLian decoction, these changes are associated with changes in *A. muciniphila* (Deng et al., 2021). *Coptis chinensis* is a Chinese herb that can improve the intestinal barrier by increasing the abundance of *Akkermansia* (Yang et al., 2023; Zhao et al., 2023). Codonopsis pilosula extract could alleviate the symptoms of acute colitis in mice by regulating the intestinal microbiota. Bacterial 16S rRNA sequencing analyzed showed that addition of Codonopsis pilosula extract stimulating the growth of three important probiotics, *Bifidobacterium* spp.*, Lactobacillus* spp.*, and A. muciniphila.* Gas chromatography determined the content of SCFAs in feces found that Codonopsis pilosula extract has selectively increased the bacteria that produced SCFAs, and promoted the production of SCFAs, alleviated malnutrition symptoms in colitis (Jing et al., 2018). These findings imply that the administration of herb medicine can ameliorates the symptoms of acute colitis.

The relationship between *A. muciniphila* and herb medicine play an important role in maintaining intestinal barrier integrity and intestinal microenvironment homeostasis. More animal trials combined with clinical studies are urgently needed to further elucidate the mechanisms for the effect of specific probiotic bacteria in preventing IBD.

### 4.2 *A. muciniphila*, herbal medicine, and human immunodeficiency virus

The CC chemokine receptor 5 is an attractive target for HIV as it is expressed at high levels in intestinal CD4 T cells, which are severely depleted during infection (Brenchley and Douek, 2008; Weichseldorfer et al., 2022). HIV infection disrupts the intestinal barrier, leading to translocation of microbial products. Although antiretroviral therapy (ART) can control the viral load and CD4 T cell count, it is difficult to normalize gut dysbiosis and chronic immune activation, which affect disease progression (Marchetti et al., 2013; Zicari et al., 2019). Evidence has shown that gut damage and microbial translocation contribute to the risk of non-AIDS comorbidity and mortality (Ouyang et al., 2023). Therefore, upregulation of intestinal barrier function may be a promising strategy in people living with HIV (PLWH).

It is particularly important to explore the gut microbiota mechanisms in PLWH (Schretter, 2020). A reduction in *A. muciniphila* in the gut is one of the changes associated with ART-naïve and ART-treated PLWH compared with noninfected people (Rocafort et al., 2019). Furthermore, the abundance of *A. muciniphila* in 27 chronically HIV-1-infected patients treated with ART was similar to the abundance in noninfected people (Rocafort et al., 2019). A Phase 2b trial studying the ability of supplementation with probiotics to reduce disease-associated systemic immune activation in an immune-unresponsive phenotype of PLWH showed that 18 patients in the probiotic group had increased blood CD8 and CD4+ T cell activation compared with 10 patients in the placebo group (Rousseau et al., 2022).

A study of seven chronically simian immunodeficiency virus-infected pigtail macaques showed that ART plus probiotics/prebiotics increases the frequency and functionality of the gastrointestinal tract by upregulating the expression of APC-related genes, enhancing the reconstitution and functionality of CD4+ T cells, and reducing the fibrosis of colonic lymphoid follicles (Klatt et al., 2013). Thus, ART combined with probiotic/prebiotic symbiotic supplementation in PLWH may promote increased intestinal CD4+ T cell reconstitution and mitigate inflammatory sequelae, significantly improve disease prognosis, and provide a new perspective for the management of HIV (Klatt et al., 2013; Ortiz et al., 2016).

Herbal medicine regulates the intestinal flora, inhibits the abnormal proliferation of opportunistic pathogens, and delays the clinical progression of AIDS. 16S rRNA gene sequencing of 30 patients with post-ART immunodeficiency who were treated with Shenlingguben immune granules or Artesunate tablets plus ART showed that treated patients possessed a higher abundance of *Sutteralla* species and Verrucomicrobiota after treatment, which was positively correlated with enhancements in immune function and the CD4+ T cell count (Wu, 2021). In a mouse model of immunosuppression established by cyclophosphamide, the administration of dandelion and *Codonopsis* significantly improved the immune organ index, immunoglobulin levels, and the white blood cell count, possibly in association with the abundance of *Bifidobacterium* and *Lactobacillus* and an increase in intestinal flora diversity (Gu et al., 2019). The relationship between *A. muciniphila* abundance and herbal medicine in PLWH requires further elucidation.

### 4.3 *A. muciniphila*, herbal medicine, and cancer immunotherapy

Cancer immunotherapy is an innovative treatment and its effectiveness depends on the activity of the host immune system. The intestinal microbiota plays an important role in immune regulation, the immune response, and tumor immunity by affecting the tumor microenvironment. Disturbance of the intestinal microbiota promotes tumor formation. In addition, the microbiota may play an important role in ameliorating tumorigenesis (Janney et al., 2020; Wang et al., 2020; Matson et al., 2021; Schmitt and Greten, 2021). The intestinal microbiota is closely associated with various immune cells. Some bacteria, such as *A. muciniphila*, Clostridiales, and *Ruminococcaceae*, have been shown to prevent systemic immune suppression by strengthening intestinal barrier integrity and systematically reducing inflammation. Furthermore, the microbiome governs the gut ecosystem to circumvent primary resistance to immune checkpoint inhibitors (Routy et al., 2018).

One study investigated whether the gut microbiome affects the response to anti-PD-1 immunotherapy in patients with hepatocellular carcinoma using metagenomic sequencing. The results showed that the intestinal microbiome, specifically *A. muciniphila* and *Ruminococcaceae* spp., can improve the efficacy of PD-1 by enhancing immune metabolism during the treatment of hepatocellular carcinoma. In addition, changes in the abundance of the gut microbiome may provide an early prediction of the outcome of immunotherapy in hepatocellular carcinoma (Zheng et al., 2019), and the long-term survival prospects (Kharofa et al., 2023). Analysis of the fecal metagenome in long-term survivors of pancreas adenocarcinoma showed that patients cured of this cancer had a greater abundance of *A. muciniphila*, compared with patients who completed pancreatectomy and chemotherapy (Kharofa et al., 2023). Amuc_2172, a newly discovered antitumor component of *A. muciniphila*, inhibits the viability of cells by promoting the cytotoxic T lymphocyte-related immune response (Jiang et al., 2023). A subcutaneous melanoma and colorectal tumor-bearing mouse model showed that IL-2 combined with the oral administration of *A. muciniphila* strengthens antitumor immune surveillance by activating the Toll-like receptor 2 signaling pathway (Shi et al., 2020a). These findings propose that *A. muciniphila* in cancer treatment, is a novel therapeutic strategy with prospecting application for cancer treatment in clinical practice.

The active ingredients of herb medicine or compound interact with intestinal flora on target organs to enhance immunity against tumors. A recent attempt to evaluate the effects of sini Decoction(SND) and gut microbes on colorectal cancer revealed that SND upregulates the expression of CD8+ T lymphocytes, increases the relative contents of beneficial bacteria (including *A. muciniphila*), and ameliorates the degree of malignancy of the tumor, which demonstrates that SND changes the intestinal microbiota composition in mice (Wang et al., 2021). Huoxue Yiqi Recipe-2 is a classic herbal medicine prescription described in the “Synopsis of Prescriptions of the Golden Chamber”, which increases the abundance of *A. muciniphila* and may therefore enhance the therapeutic effect of PD-L1 (Teng et al., 2020). Wenzi Jiedu Recipe (WJR) has been proven to be clinically useful in the treatment of colorectal cancer. The 16S rDNA sequencing method was used to analyze the changes of gut microbes revealed that WJR significantly enriched for *Oscillibacter and Bacteroides_acidifacien* in tumor-bearing mice with colorectal cancer. Meanwhile, WJR significantly increased the proportion of CD8+ T cells and the expression of immune-associated cytokines IL-10, IFN-γ, and TNF-α (Qiu et al., 2022). The above studies suggest that traditional Chinese medicine can improve tumor immunotherapy by adjusting the intestinal microecological structure. It is anticipated that clinical outcomes for patients with cancer will improve in the near future with the introduction of cancer immunotherapy combined with *A. muciniphila*.

Recently, medical researchers have proved that herb medicine can enrich intestinal bacteria and enhance immunity. However, the relationship between intestinal flora and TCM syndrome type in immunotherapy needs further research.

### 4.4 *A. muciniphila*, herbal medicine, and immune-related liver injury

The liver is a key, frontline immune tissue that maintains the homeostasis of the systemic immune response and overall tissue health (Li et al., 2015). Immune-related liver injury(IRLI) is mediated by the immune response and characterized by inflammatory cell infiltration, inflammatory granuloma formation, and damage to the structure of the hepatocyte cords (Nakano et al., 2010). IRLI is an important factor in the development of liver fibrosis, cirrhosis, and even liver tumors, and determines the outcome of the disease (Zhan and An, 2010). Various lines of evidence have linked gut microbiota dysbiosis with barrier autoimmunity and beyond, especially in the setting of immune-related liver injury (Abe et al., 2018; Wei et al., 2020). The gut microbiota and harmful substances initiate the downstream immune signal of liver cells through toll-like receptor 4 and other pattern recognition receptors, directly cause an inflammatory response, thereby aggravating inflammation-induced liver injury (Hritz et al., 2008).

Accumulating evidence indicates that the severity of immune-mediated liver injury is related to the microbiome. Dysbiosis is the cause of the development of immune hepatitis. *Klebsiella and Enterococcus* are up-regulated and *bifidobacteria, ruminococcus and Lactobacillus* are down-regulated in patients with immune hepatitis, which leads to a decrease in the ratio of *bifidobacteria to Enterococci* (Liu et al., 2022). An animal model of acute liver injury was induced by the injection of concanavalin A into the tail vein. Administration of *A. muciniphila* decreased hepatocellular apoptosis and the concentrations of pro-inflammatory cytokines and chemokines. This may be because the decreased concentrations of Fas, DR 5, and CB1 receptor enhanced the expression of Bcl-2, occludin, and Tjp-1 (Wu et al., 2017). Amuc_1100 (Amuc) may exert an immunomodulatory function by upregulating the mRNA levels of Nlrp3 and Asc in the liver of *S. typhimurium*-infected mice (Shi et al., 2020b; Song et al., 2023)

In concanavalin A-treated mice, the administration of zhenqiyigan decoction results in decreased serum concentrations of ALT, AST, and superoxide dismutase, increased expression levels of Fas, FasL, Bax, and PCNA, and regulates the balance of Thl/Th2, indicating that zhenqiyigan decoction plays a protective role in promoting the apoptosis of damaged hepatocytes and stimulating hepatocyte regeneration (Xiaoli, 2016).A study found that geniposide and chlorogenic acid, the active ingredients of Qushi Huayu decoction, can reduce the expression of genes required for lipid synthesis in the liver of rats with non-alcoholic fatty liver disease, and reduce serum LPS levels in rats. This may be because the increased abundance of Bacteroides and *Clostridium* induces Treg cell production that inhibits intestinal inflammation and improves intestinal barrier function in rats (Feng et al., 2017).

Research on the regulation of gut microbiota dysbiosis in IRLI by herb medicine is well underway, which will provide a novel direction for targeting the gut microbiota to explore potential therapeutic strategies for IRLI. However, more studies are still at the efficacy observation stage and fail to address the in-depth mechanism.

## 5 Mechanisms of action of *A. muciniphila* and herbal medicine in immune-related diseases

**Figure 1:**
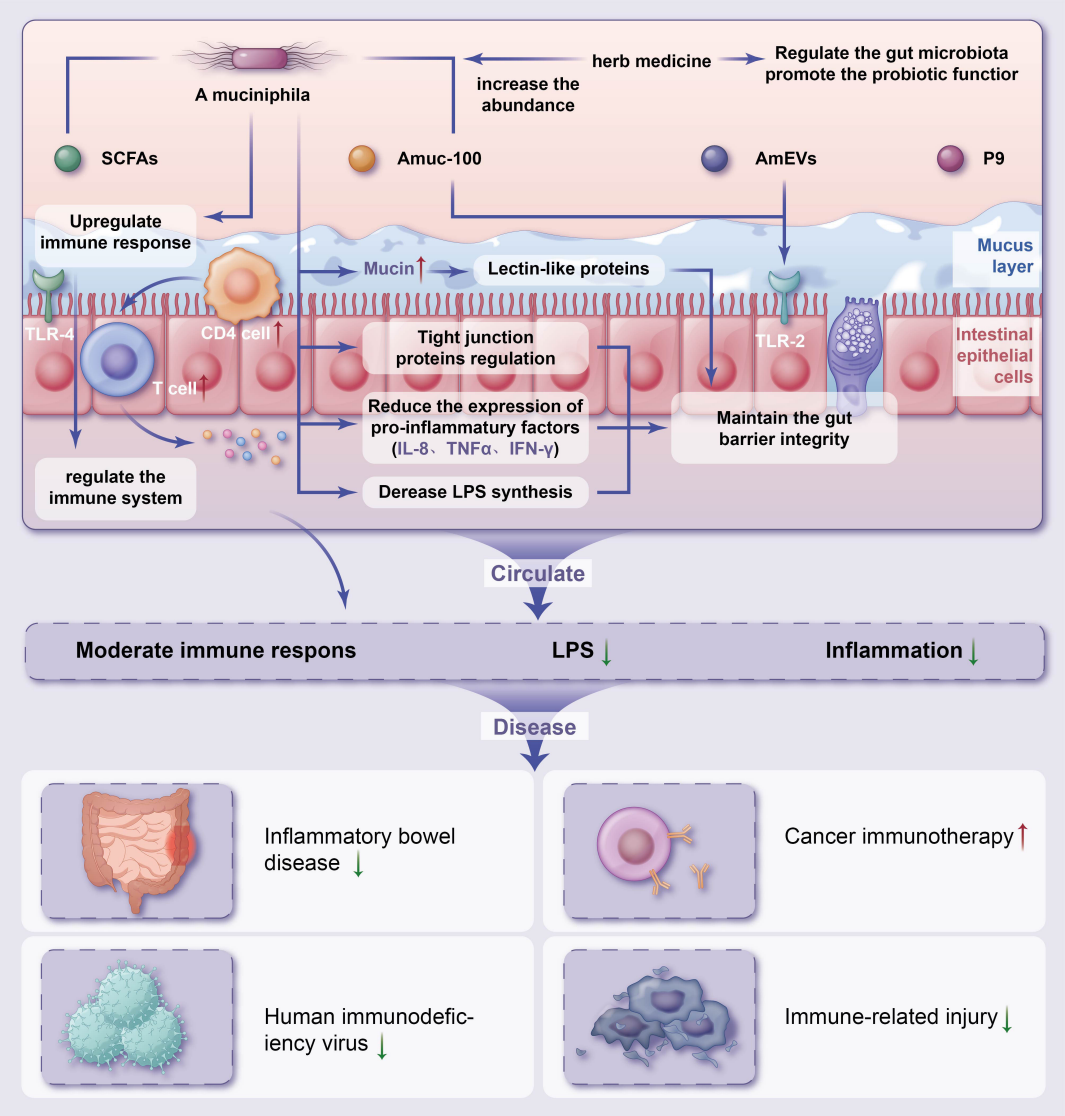
Potential mechanisms of herb medicine and action of *Akkermansia muciniphila* in immune-related diseasesThe potential mechanisms of herb medicine and action of *A. muciniphila* in controlling IRDs are (1) regulate the gut microbiota promote the probiotic functor and reduce chronic inflammation (2); intestinal homeostasis are associated with increased numbers of goblet cells, enhanced mucus barrier (3); *A. muciniphila* derived extracellular vesicles (AmEVs), a bilayer structure composed of lipid, protein, lipopolysaccharides as well as other molecules, and Amuc_1100 also regulate intestinal barrier permeability by altering tight junction protein expression through activating TLR2 pathway (4); maintenance of gut barrier integrity through the reduction of LPS synthesis and reduce the express of pro-inflammatory factors (5); increase the differentiation of Tregs in CD4+ T cell population, reduce chronic inflammation and upregulate TLR4-mediated immune response, and regulate the immune system. IRDs, immune-related diseases; SCFAs, short-chain fatty acids; LPS, lipopolysaccharide; IL, interleukin; TNF, tumor necrosis factor; IFN, interferon; TLR, Toll-like receptor.

A large body of experimental and clinical evidence has accumulated on the use of gut microbiota strategies to improve health. Several models have been proposed based on the available evidence for the mechanisms underlying the effects of *A. muciniphila* in the treatment of IRDs (**Figure 1**).

### 5.1 Maintenance of the intestinal barrier

The gut microbiota exerts a number of functions including preventing epithelial damage, maintaining the integrity of the intestinal barrier, and preventing pathogens from invading the mucosal tissues. The intestinal mucous layer plays an important role in protecting against mechanical, chemical, and biological attack, and contributes to maintaining a steady state (Cornick et al., 2015; Etienne-Mesmin et al., 2019). The mucus layer can directly attach to lectin-like proteins by immune cells as a result of the glycan immune effect. Mucus is part of the innate intestinal mucosal barrier that reduces the exposure of antigens and bacteria to the intestinal epithelial cell-based immune system, and its protective effect is also due to its synergistic action with the immune system to serve as the first line of immune defense against potentially harmful compounds (Pelaseyed et al., 2014; Corfield, 2015; Johansson and Hansson, 2016). The gut microbiota is important in the formation and regulation of the intestinal mucus layer. The mucosal surface is enriched in *A. muciniphila*, which protect against pathogen adhesion by increasing mucus production and occupying available binding sites on the mucins, thus preventing pathogen invasion (Kim and Ho, 2010; Donaldson et al., 2016). Probiotics might also affect the mucus barrier. *A. muciniphila* is a key bacterium that modifies the mucus layer to communicate with host cells and stimulates the production of mucus (Everard et al., 2013; Shin et al., 2014; Wu et al., 2017; Alvarado et al., 2019; Bárcena et al., 2019; van der Lugt et al., 2019). *A muciniphila* expresses specific outer membrane proteins, thus potentially strengthening the intestinal barrier (Paone and Cani, 2020). *A. muciniphila*-derived extracellular vesicles influence gut permeability through the regulation of tight junctions (Chelakkot et al., 2018). A recent study revealed that after gavage of *A. muciniphila* into Apc^Min/+^ mice, the thickness of the intestinal mucus layer returned to normal, accompanied by an increase in the number of *A. muciniphila* in the mucus layer and goblet cells, which might be related to changes in the intestinal immune system, permeability, and microbial metabolites caused by *A. muciniphila* colonization (Dingemanse et al., 2015). Similarly, a study found that supplementation with *A. muciniphila* improved glucose sensitivity, inflammation, antioxidant capacity, and intestinal barrier function in mice (Ma et al., 2023). Herbal medicine has been shown to be involved in the repair of epithelial barrier integrity by upregulating the expression of the tight junction protein zonula occludens protein 1 (ZO-1) and occludin contents (Alharris et al., 2022). This implies that *A. muciniphila* may exert a beneficial regulatory role in host immune function by protecting the intestinal barrier. Herbal medicine can increase the thickness of the mucus layer, the expression of tight junction proteins, and the population of *A. muciniphila* in the intestine. Meanwhile, supplementation with herbal medicine reduced endotoxemia and the expression of various pro-inflammatory factors in mice (Zhu et al., 2018). Therefore, it is reasonable to believe that there is a crucial link between *A. muciniphila* and herbal medicine that maintains the integrity of the intestinal barrier.

### 5.2 Adjusted microbiome

Disorders of the gut microbiota increase intestinal permeability and lead to systemic inflammation by activating immunity, ultimately causing disease. Oral administration of *A. muciniphila* significantly decreased inflammatory cell infiltration, the mRNA expression of inflammatory factors, and improved dysbiosis of the intestinal flora in mice (Mulhall et al., 2022; Zhang et al., 2022a). Analysis of 16S rRNA amplicon sequences showed that *Akkermansia* induced significant intestinal microbiota alterations, including the increased abundance of *Akkermansia*, *Muribaculaceae*, and *Parabacterbides goldsteinii*, and the decreased abundance of *Escherichia_Shigella* and *Enterobacteriaceae* (Xu et al., 2023). Proteomic and metabolomic analyses revealed that *A. muciniphila* activates glucose and lipid metabolism in gut epithelial cells, leading to an increase in ATP production (Song et al., 2021). Herbal medicine acts through a variety of immunomodulatory pathways to suppress inflammation, including modulating microbial composition and reducing intestinal inflammation and permeability (Ma et al., 2018; Chhabra et al., 2021; Alharris et al., 2022; Medina-Larqué et al., 2022; Lu et al., 2023). Andrographolide prevents type 2 diabetes by modifying the gut microbiota composition, elevating the Bacteroidetes/Firmicutes ratio, enriching microbial species such as *A. muciniphila*, and increasing the short-chain fatty acid concentration (Su et al., 2020). Ootheca mantidis, a commonly prescribed herbal medicine for chronic kidney disease, mitigates renal fibrosis in mice by modulating glutamine metabolism, remodeling the gut microbiota, increasing the levels of some probiotics (including *A. muciniphila*), and downregulating apoptosis and inflammation-associated pathways (Wang et al., 2023). Dysbiosis of the gut microbiome and related metabolites has been intimately associated with disease. In a 5/6 nephrectomized rat model, 16S rRNA sequencing and untargeted metabolomic analysis showed a marked decline in microbial diversity and richness, accompanied by significant changes in 291 serum metabolites, which were mediated by altered enzymatic activities and dysregulation of lipids, amino acids, bile acids, and polyamine metabolism. Administration of poricoic acid A and Poria cocos ameliorated microbial dysbiosis and lowered the serum levels of microbial-derived products, including glycine-conjugated compounds and polyamine metabolites (Feng et al., 2019). Sodium houttuyfonate (SH), a derivative of the medicinal herb Houttuynia cordata Thunb, could maintain gut microbiota homeostasis, thereby improving intestinal function. Administration with SH weaken the oxidative stress and inflammatory response and enhance the intestinal mucosal integrity in mice model. 16S rRNA gene sequencing results showed that SH regulate the abundance and diversity of microbiota with an increase of beneficial bacteria, including SCFAs producing bacteria and probiotics (Cheng et al., 2023). Taken together, research shows that herbal medicine might have beneficial biological effects on *A. muciniphila*, which plays an important therapeutic role by regulating the population, distribution, and metabolites of intestinal microbiota to promote its probiotic function.

### 5.3 Enhancement of immune function

*A. muciniphila* strains increase the differentiation of Tregs from CD4+ T cell populations and alleviate chronic inflammation by reducing the concentrations of IL-8, TNF-α, and IFN-γ through the enhancement of anti-inflammatory Tregs (Zhai et al., 2019). Toll-like receptor 4 functions as a sensor mediating the crosstalk between the intestinal commensal microbiome and host immunity. A study exploring the relationship between Toll-like receptor 4 and intestinal microbial ecology found that *A. muciniphila* ameliorated colitis by upregulating the immune responses mediated by RORγt+ Treg cells (Liu et al., 2022). Butyrate serves as an energy source for intestinal epithelial cells and has anti-inflammatory effects. *A. muciniphila* is beneficial in maintaining the homeostasis of the intestinal microbiome by producing butyrate, thus promoting anti-inflammatory immune functions (Huang et al., 2022). A study found that the therapeutic outcome of IL-2 was significantly potentiated when *A. muciniphila* was increased in mice with subcutaneous melanomas and colorectal tumors. Mechanistically, Amuc, derived from the outer membrane protein of *A. muciniphila*, activates the Toll-like receptor 2 signaling pathway, which is conducive to the antitumor immune response (Shi et al., 2020a). Although research shows that *A. muciniphila* regulates the immune system, the mechanisms underlying the effects of *A. muciniphila* on the regulation of anti-inflammatory immune functions require further investigation.

Herbal medicines can improve the body’s immune response by indirectly exerting an immune regulation effect or directly acting on the intestinal epithelial cells (Xu et al., 2015). Yi-Yi-Fu-Zi-Bai-Jiang-San (YYFZBJS) is a classical prescription that can regulate T lymphocytes and improve immunity. Diversity analysis of fecal samples demonstrated that YYFZBJS regulated animal’s natural gut flora, including *Bacteroides fragilis, Lachnospiraceae* and so on. Intestinal lymphatic, and mesenteric lymph nodes, accumulated CD4+ CD25+ Foxp3 positive Treg cells were reduced by YYFZBJS in Apc Min/+ mice. In conclusion, YYFZBJS regulate inflammation expression by the gut microbiota mediated immune cells and increased immune function (Sui et al., 2020). GeGen QinLian decoction (GQD), a Chinese herb formula. 16S rRNA sequencing revealed that GQD can restore the intestinal flora, resulting in an increase in *A. muciniphila*, *Desulfovibrio_C21_c20* and *Lactobacillus_salivarius*, and a decrease in *Escherichia_coli.* GQD inhibited the NOD/RIP2/NF-κB signaling pathway in the intestine and affected the expression of downstream related inflammatory cytokines in mesenteric lymph nodes and serum in mouse. In addition, GQD treatment showed systemic protection by restraining the inflammatory differentiation of CD4 + T cells. In conclusion, GQD can affect systemic immunity by restore intestinal flora (Deng et al., 2021).

In summary, herb medicinal can positively regulate the intestinal microorganisms, promotes probiotic colonization, inhibit the growth of pathogenic microorganisms, influence the differentiation and apoptosis of intestinal cells, thereby improve the intestinal barrier function and immune function. However, the intestinal microbiota is a dynamically changing and complex population. In-depth research is needed in the study of herbal medicines acting on the gut flora to modulate the immune system for the treatment of IRDs.

## 6 Conclusions and perspectives

When the intestinal barrier is destroyed, the permeability of intestinal mucosa increases, bacterial translocation causes systemic inflammatory response, while the occurrence and development of IRDs are mostly closely related to the inflammatory response of the body. *A. muciniphila* plays an important role in protecting the intestinal barrier. It is crucial to understand the link between *A. muciniphila* and IRDs. The abundance of *A. muciniphila* may be reduced by the interaction between the organism and the gut microbiota when the host’s immune function is suppressed. *A. muciniphila* has a complex relationship with the immune responses of the host and is a potential therapeutic target for IRDs linked to the microbiome. *A. muciniphila* also exerts probiotic properties and has been used in therapeutic interventions with satisfactory results. The mechanism of action of herbal medicine in treating IRDs is associated with repairing the intestinal barrier, regulating the intestinal microbiota and its metabolites, or regulating the immune response to alleviate disease. Using herb medicine regulating the gut microbiota to maintain it in a relatively stable state has great potential and clinical research value in the treatment of IRDs. However, several key issues remain unresolved. Most of the mechanistic studies on the related effect of *A. muciniphila* and herb medicine have been performed using animal models. Given the differences in the genetics and external environment between animal models and humans, the mechanisms of action of *A. muciniphila* in humans are yet to be proven. There is an urgent need for more animal trials combined with clinical studies to further elucidate the mechanistic basis for the effects of *A. muciniphila* in the treatment of IRDs and to develop novel therapeutic targets. Future studies should focus on how the active components of herbal medicine are metabolized by intestinal flora, and whether these metabolites have synergistic or antagonistic effects on the treatment of autoimmune diseases, this will enable the discovery of new beneficial metabolites of the intestinal flora, which will provide a new direction for clinical drug treatment of IRDs.

## Author contributions

XD: Writing – original draft. P-FM: Conceptualization, Writing – review & editing. X-XM: Conceptualization, Writing – review & editing. J-YY: Conceptualization, Writing – review & editing. L-PL: Conceptualization, Writing – review & editing. L-RX: Writing – review & editing.
